# Deep learning predicts cervical lymph node metastasis in clinically node-negative papillary thyroid carcinoma

**DOI:** 10.1186/s13244-023-01550-2

**Published:** 2023-12-20

**Authors:** Li-Qiang Zhou, Shu-E. Zeng, Jian-Wei Xu, Wen-Zhi Lv, Dong Mei, Jia-Jun Tu, Fan Jiang, Xin-Wu Cui, Christoph F. Dietrich

**Affiliations:** 1 Sino-German Tongji-Caritas Research Center of Ultrasound in Medicine, Department of Medical Ultrasound, Tongji Hospital, Tongji Medical College, Huazhong University of Science and Technology, No. 1095, Jiefang Avenue, Wuhan, Hubei Province 430030 China; 2 MOE Frontiers Science Center for Precision Oncology, Faculty of Health Sciences, University of Macau, Macau, SAR 999078 China; 3 Department of Ultrasound, Hubei Cancer Hospital, Tongji Medical College, Huazhong University of Science and Technology, Wuhan, Hubei Province China; 4 Department of Ultrasound, First Affiliated Hospital of Zhengzhou University, Zhengzhou, China; 5 Department of Artificial Intelligence, Julei Technology Company, Wuhan, China; 6 Department of Medical Ultrasound, Wuchang Hospital affiliated with Wuhan University of Science and Technology, Wuhan, China; 7 Department of Medical Ultrasound, Wuhan Hospital of Traditional Chinese and Western Medicine, Tongji Medical College, Huazhong University of Science and Technology, Wuhan, China; 8 Department of Ultrasound, The Second Affiliated Hospital of Anhui Medical University, Hefei, China; 9 Department of Allgemeine Innere Medizin, Kliniken Hirslanden Beau Site, Salem und Permanence, Bern, Switzerland

**Keywords:** Deep learning, LN metastasis prediction, Papillary thyroid cancer, US diagnosis

## Abstract

**Objectives:**

Precise determination of cervical lymph node metastasis (CLNM) involvement in patients with early-stage thyroid cancer is fairly significant for identifying appropriate cervical treatment options. However, it is almost impossible to directly judge lymph node metastasis based on the imaging information of early-stage thyroid cancer patients with clinically negative lymph nodes.

**Methods:**

Preoperative US images (BMUS and CDFI) of 1031 clinically node negative PTC patients definitively diagnosed on pathology from two independent hospitals were divided into training set, validation set, internal test set, and external test set. An ensemble deep learning model based on ResNet-50 was built integrating clinical variables, BMUS, and CDFI images using a bagging classifier to predict metastasis of CLN. The final ensemble model performance was compared with expert interpretation.

**Results:**

The ensemble deep convolutional neural network (DCNN) achieved high performance in predicting CLNM in the test sets examined, with area under the curve values of 0.86 (95% CI 0.78–0.94) for the internal test set and 0.77 (95% CI 0.68–0.87) for the external test set. Compared to all radiologists averaged, the ensemble DCNN model also exhibited improved performance in making predictions. For the external validation set, accuracy was 0.72 versus 0.59 (*p* = 0.074), sensitivity was 0.75 versus 0.58 (*p* = 0.039), and specificity was 0.69 versus 0.60 (*p* = 0.078).

**Conclusions:**

Deep learning can non-invasive predict CLNM for clinically node-negative PTC using conventional US imaging of thyroid cancer nodules and clinical variables in a multi-institutional dataset with superior accuracy, sensitivity, and specificity comparable to experts.

**Critical relevance statement:**

Deep learning efficiently predicts CLNM for clinically node-negative PTC based on US images and clinical variables in an advantageous manner.

**Key points:**

• A deep learning-based ensemble algorithm for predicting CLNM in PTC was developed.

• Ultrasound AI analysis combined with clinical data has advantages in predicting CLNM.

• Compared to all experts averaged, the DCNN model achieved higher test performance.

**Graphical Abstract:**

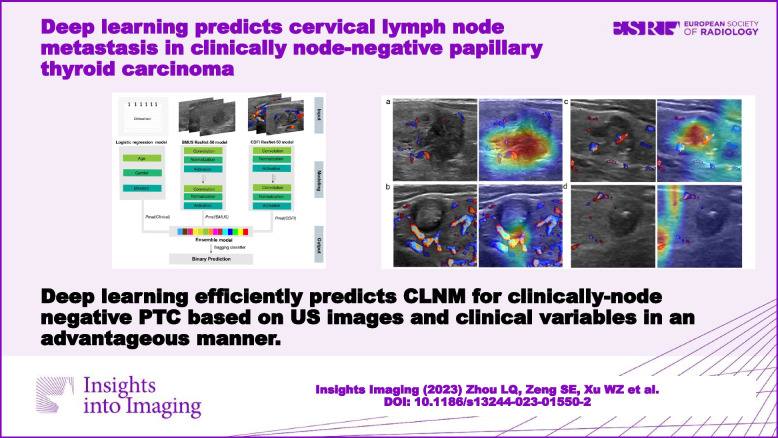

**Supplementary Information:**

The online version contains supplementary material available at 10.1186/s13244-023-01550-2.

## Background

As one of the most frequently diagnosed cancers worldwide, the incidence rate of thyroid cancer continues to increase substantially over the past two decades [1]. Papillary thyroid cancer (PTC), the most universal malignancy of the thyroid, is frequently prone to metastasis with a high risk of invasion, especially involving cervical lymph node metastasis (CLNM), which can reach an incidence of 40–90% [2]. CLNM as a high-risk factor of death and recurrence is closely associated with the pathologic staging, prognosis, and guidance of treatment. Therefore, the challenge faced by physicians is to undertake an appropriate diagnostic work-up and then balance the therapeutic approach for patients with thyroid cancer. Generally, ultrasound (US)-guided fine needle aspiration cytology (FNAC) or intraoperative CLN inspection is the definitive gold standard for determining CLNM, but it may cause postoperative complications due to its invasiveness and has a low sensitivity and specificity for clinically node-negative thyroid cancer [3].

Preoperative imaging evaluation is extremely valuable due to its convenient, comprehensive, and non-invasive properties. As the first imaging methodology recommended by the American Thyroid Association (ATA), US exhibits superior advantages compared with computed tomography (CT) in detecting CLNM [4]. However, it is difficult to evaluate the deep anatomy located in the central cervical compartment with sufficient satisfactory sensitivity, which leads to a great number of missed CLNM [5]. Moreover, CLN with micro-metastasis will further confuse real diagnosis results due to the false negative US characteristics [6]. It is worth noting that there are plenty of available sonographic features of primary thyroid cancer correlated with CLNM. For example, the size and number of primary tumor, ill-defined tumor edge, and the coexistence of Hashimoto’s thyroiditis (HT) were reported as independent predictive factors for the state of CLN [7, 8]. The presence of capsule invasion, micro-calcifications, and internal vascularity also have impacts on CLNM [9, 10]. Besides, enhanced stiffness of primary tumor tested by US-based elastography technique of shear wave elastography (SWE) and acoustic radiation force impulse (ARFI) were quantitatively instrumental to predict CLNM in thyroid cancer patients [11, 12]. Consequently, several studies have attempted to develop validated nomograms using multivariable logistic regression models to predict and quantify the likelihood of CLNM based on preoperative clinical and radiological findings, but the method of traditional machine learning showed unsatisfactory results due to low discrimination ability and absent reproducibility in validations [13–15]. In addition, the cross-sectional design used in the above studies cannot determine the causal relationship between risks and variables and also artificially limits the risk factors of CLNM.

Deep learning algorithms, especially deep convolutional neural network (DCNN), exhibit obvious advantages in recognizing image details. Compared with traditional machine learning algorithms that mainly depend on pre-defined features, deep learning algorithms can automatically and quantitatively evaluate complex medical image features and obtain powerful image recognition capabilities, thereby achieving higher diagnostic accuracy [16]. Although deep learning technologies have been extensively exploited in terms of diagnosis, prognosis, and treatment response in oncology due to their fast, accurate, and reproducible advantages after specific training, there are very few studies focusing on lymph node metastasis prediction based on medical images of thyroid cancer in the literature [17, 18].

Previous study has demonstrated the feasibility of deep learning to diagnose the metastasis of lymph node using US images [19]. In this work, we attempt to explore the feasibility of deep learning models to predict CLNM through preoperative US images (B-mode US (BMUS), color Doppler flow imaging (CDFI)) of thyroid cancer nodules, and clinical variables of primary thyroid cancer. As far as we know, it is the first deep learning system for automatically predicting CLNM from primary thyroid cancer US images. It is shown that the proposed deep learning neural network achieves better prediction performance than radiologists. The US imaging-based CLNM predictions are of great significance in the clinic due to a series of US features closely related to CLSM, such as microcalcification, blurred edges, and abundant blood flow, so they can help achieve precise medical practices and tailored clinical treatment.

## Methods

### Study design and datasets

Ethical approval was obtained from the Institutional Review Board of Tongji Medical College of Huazhong University of Science & Technology for this retrospective analysis, and the informed consent requirement was waved (approval number: 2019S876). Two independent datasets were analyzed containing histologically confirmed PTC patients undergoing surgical resection but had clinically negative lymph nodes (physical examination or imaging (CT/MRI)). We firstly developed and validated deep learning models for internal test in primary dataset A. The generalizability and further external test of the neural network was then evaluated on secondary dataset B. In addition, expert’s evaluations were also investigated to compare the predictive performance with deep learning. All the included thyroid cancer patients aged 18 years or older and underwent preoperative FNAC evaluation of cervical LN, thyroid surgery, and CLN pathological evaluation. All thyroid cancer patients performed CND. Thyroid cancer confirmed to be at least N1b by preoperative evaluation or intraoperative frozen section concurrently underwent lateral lymph node dissection. The inclusion criteria are as follows: (1) pathologically confirmed primary PTC with clinically negative lymph nodes, (2) available preoperative US images including BMUS and CDFI, (3) image quality was sufficient for analysis, and (4) no treatment prior to surgical treatment. All thyroid US images were collected from the thyroid US imaging database of the two hospitals and stored in DICOM format at their original resolution. Pathological examinations of thyroid and CLN were assessed by board-certified pathologists of each hospital according to internationally harmonized classification standards. Some patients have performed multiple thyroid US examinations, and only the most recent ones before surgery were included. For each patient, the most typical BMUS and CDFI images were filtered by several US radiologists from Tongji Hospital and Hubei Cancer Hospital for image quality control. For section images that are repetitive, blurry, too large or too small in scope, and cannot be diagnosed as malignant by radiologists, they will be screened out. US equipment manufactured by Philips (Amsterdam, the Netherlands; L12-5, VL 13–5, and L18-5) and GE Healthcare (Pittsburgh, PA; LOGIQ S8, 7, E9) were utilized to produce the US images.

### Deep neural network

**Fig. 1:**
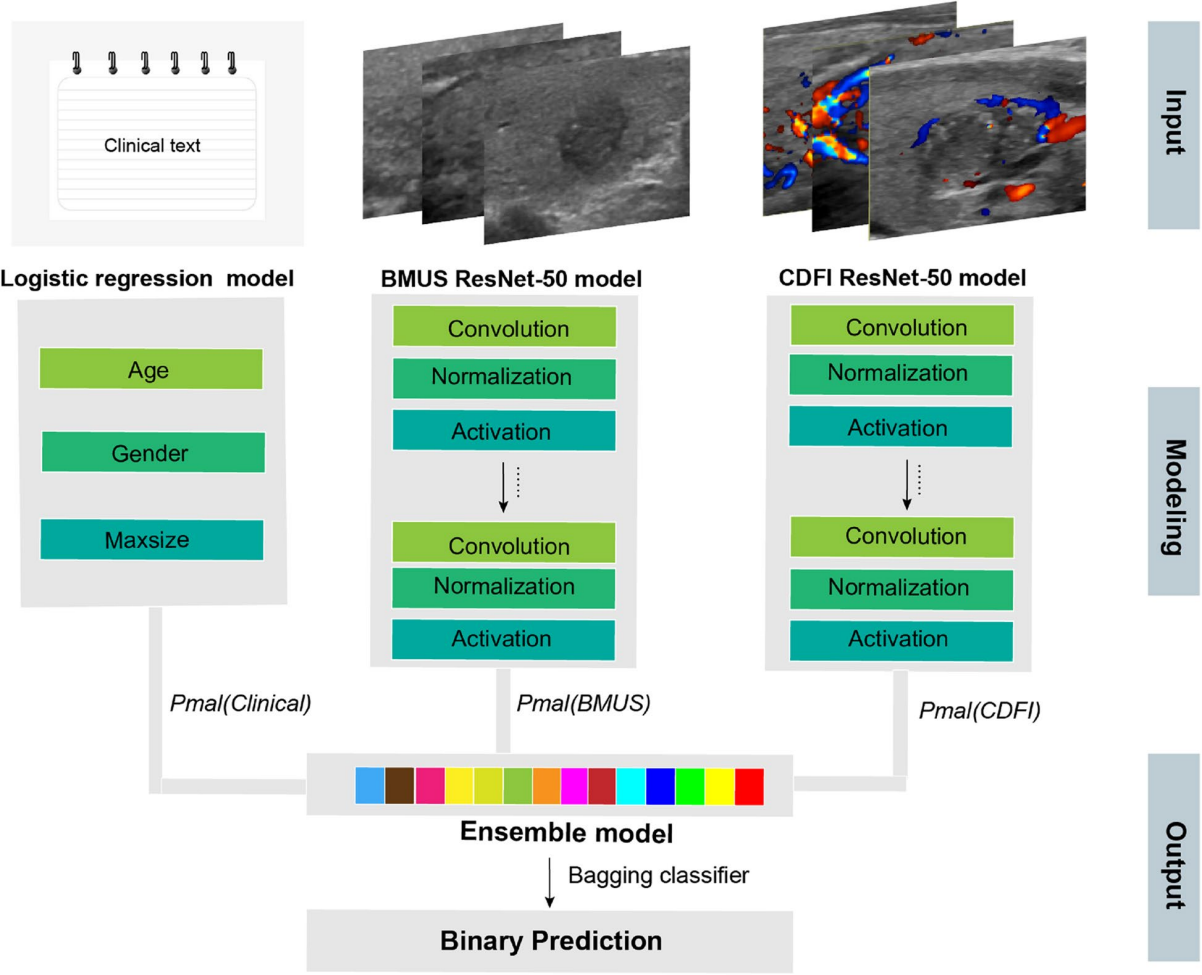
An illustration of the ensemble model architecture

The ResNet-50 DCNN model was separately trained on BMUS and CDFI images [20]. To train the network, we utilize stochastic gradient descent as the optimizer, binary cross-entropy as the loss function, and binary accuracy as the metrics function, with a learning rate of 0.001, a batch size of 32, and 300 epochs. During the training process, all the images were uniformly sized to 225 × 225 pixel squares using bilinear interpolation and then augmented by horizontal flip, vertical flip, crop, and scale transformations to increase the variability of the training set and avoid model overfitting. The weights of pre-trained CNNs from ImageNet were utilized to initialize our models’ weights and biases and remained unchanged during the training process. The trained model predicts the input data based on the mapping relationship established between the image input features (e.g., image pixels) and the corresponding output labels (e.g., metastasis or non-metastasis). The clinical variables (age, gender, and tumor maximum size) were entered into a separate model that used logistic regression to predict CLNM. Additionally, we set up a kind of ensemble model by using bagging classifiers [21] in combination with the output of the clinical variable logistic regression model, the BMUS model, and the CDFI model. After multiple training and validations on the same dataset, the model with the highest accuracy was selected as the final ensemble model. Based on the validation data, the ensemble model was calibrated using the Pratt algorithm. It does not require the calculation of transition functions and only uses the auxiliary array Next, which is the feature vector of the pattern string itself [22]. Figure 1 exhibits the architecture of the final ensemble model.

### Expert evaluation

To obtain clinical experts’ diagnosis accuracy on the test set, three certified experienced US radiologists (J.W.X., D.M., and J.J.T., with 11, 15, and 5 years of experience, respectively) independently interpreted 183 cases including internal test set A (*n* = 94) and external test set B (*n* = 89). They were blind to histopathologic results and only given clinical information and corresponding US images of each patient. The explanation consists of two parts: one is the qualitative assay based on the American College of Radiology Thyroid Imaging Reporting and Data System (ACR TIRADS) [23], and the other one is the quantitative prediction analysis of the lymph node metastasis probability (1–100%). Positive signs include tumor size greater than 4 cm, the coexistence of capsule invasion, micro-calcification, Hashimoto’s thyroiditis (HT), and internal vascularity. Each of these five features accounts for 20% of the overall scoring system. The radiologist makes a prediction of the likelihood of metastasis based on the ultrasound characteristics of each image. If the likelihood is greater than 50%, that is, there are more than three positive signs, then LN metastasis is considered to exist, and vice versa. The electronic Supplementary material describes the ACR TIRADS, the scoring system, and how to use them in detail.

### Model testing and statistical analysis

To evaluate the generalizability of the neural network model’s predictive performance, two independent datasets were employed as internal primary test A (*n* = 94) and external secondary test B (*n* = 89) for verification. The predictions of the DCNN model were compared with the pathological reports of surgically removed lymph nodes. The receiver operating characteristic (ROC) curve was created to demonstrate the predictive ability of deep learning model in discriminating lymph node metastasis. The performance of the radiologists was also marked by points on the same ROC curve, indicating their sensitivity and specificity. Areas under the ROC curve (AUCs) with 95% confidence intervals (CIs) were calculated, and the comparisons between AUCs were conducted using the method designed by DeLong et al. [24]. Additionally, the accuracy, sensitivity, specificity, positive predictive value (PPV), negative predictive value (NPV), F1 score, and Kappa value were reported for comparison between deep learning models, radiologists. *p* values less than 0.05 were considered as the threshold for significance.

### Code availability

The realization of our DCNN models was based on the Keras 2.3.1 with TensorFlow 2.0.0 as the backend [25]. All the models were trained on a computer with two NVidia 2080Ti GPUs. In order to allow other investigators to exploit their models, the codes applied for modeling and data analysis are publicly available on GitHub at https://github.com/MedicalDataAI/LNMP (ID: 8d22e54).

## Results

### Baseline characters

**Fig. 2:**
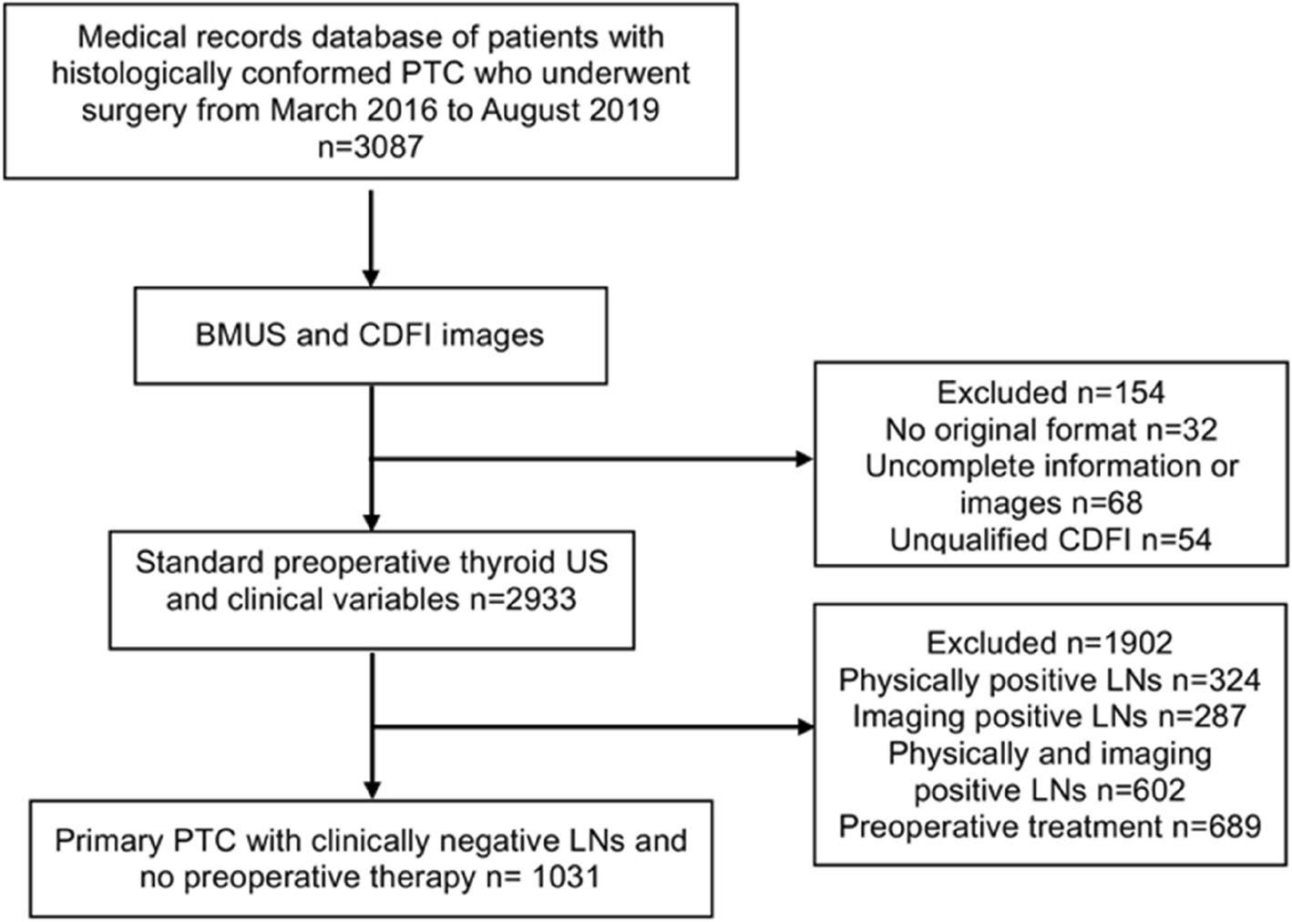
Flow chart of patient recruitment for this study. PTC, papillary thyroid cancer; BMUS, B-mode ultrasound; CDFI, color Doppler flow imaging; LNs, lymph nodes

We retrospectively collected 2062 anonymous US images of 1031 PTC as the dataset from around two hospitals in China between March 1, 2016, and August 1, 2019, including Tongji Hospital, Hubei, China (dataset A, 1884 images from 942 patients) and Hubei Cancer Hospital, Hubei, China (dataset B, 178 images from 89 patients). Figure 2 shows the patient recruitment workflow, and Table 1 demonstrates the clinical characteristics of all patients. The two independent datasets containing 1031 PTC with clinically negative lymph nodes consisted of a total of 982 images from 491 patients without CLN and 1080 images from 540 patients with CLN.

**Table 1:** Clinical characteristics of the training, validation, and test datasets

Characteristics	Training and validation sets	Test set A	Test set B
Number	848	94	89
Lymph node metastasis	400 (47.2%)	47 (50.0%)	44 (49.4%)
No lymph node metastasis	448 (52.8%)	47 (50.0%)	45 (50.6%)
Age, mean ± SD, years	42.61 ± 10.42	43.78 ± 9.27	41.53 ± 10.94
≤ 45	553 (65.2%)	55 (58.5%)	55 (61.8%)
> 45	295 (34.8%)	39 (41.5%)	34 (38.2%)
Gender			
Female	675 (79.6%)	78 (83.0%)	72 (80.9%)
Male	173 (20.4%)	16 (17.0%)	17 (19.1%)
Primary site			
Right lobe	413 (48.7%)	48 (51.1%)	44 (49.4%)
Left lobe	420 (49.5%)	43 (45.7%)	43 (48.3%)
Isthmus	15 (1.8%)	3 (3.2%)	2 (2.2%)
Metastatic site			
Central lymph node	271 (67.8%)	30 (63.8%)	28 (64.0%)
Central and lateral lymph node	89 (22.3%)	12 (24.5%)	11 (24.7%)
Lateral lymph node	40 (9.9%)	5 (11.7%)	5 (11.2%)
Prophylactic CND			
Tumor size > 4 cm	359 (42.3%)	41 (43.6%)	27 (30.3%)
Extrathyroidal extension (ETE)	125 (14.7%)	25 (26.6%)	34 (38.2%)
ETE and Tumor size > 4 cm	364 (43.0%)	28 (29.8%)	28 (31.5%)
TI-RADS category			
4A category	29 (3.4%)	4 (4.3%)	5 (5.6%)
4B category	175 (20.6%)	22 (23.7%)	22 (24.7%)
4C category	384 (45.3%)	37 (38.9%)	38 (42.7%)
5 category	260 (30.7%)	31 (32.9%)	24 (27.0%)
US size, mean ± SD, mm	12.74 ± 6.58	10.93 ± 5.91	11.47 ± 6.35

Qualitative variables are in *n* (%), and quantitative variables are in mean ± SD

### Deep learning to predict CLNM from primary thyroid cancer US images and clinical variables

To exploit deep learning as a potential solution to reliably predict CLNM, we propose developing an ensemble deep learning model trained on primary thyroid cancer BMUS, CDFI, and clinical variables, based on using pathological evaluation derived from surgical operation of CLN as labels (Fig. 2). To train, validate, and test the model, cases were gathered retrospectively from two independent general hospitals in Hubei Province, China. Dataset A from Tongji Hospital was randomly allocated to three independent cohorts: one for algorithm development (training set, 80%, *n* = 754), one for parameter optimization during algorithm development (validation set, 10%, *n* = 94), and one for algorithm testing (internal test set, 10%, *n* = 94). An additional external test was performed on dataset B from Hubei Cancer Hospital (*n* = 89).

### The ensemble DCNN model achieves the best results

**Fig. 3:**
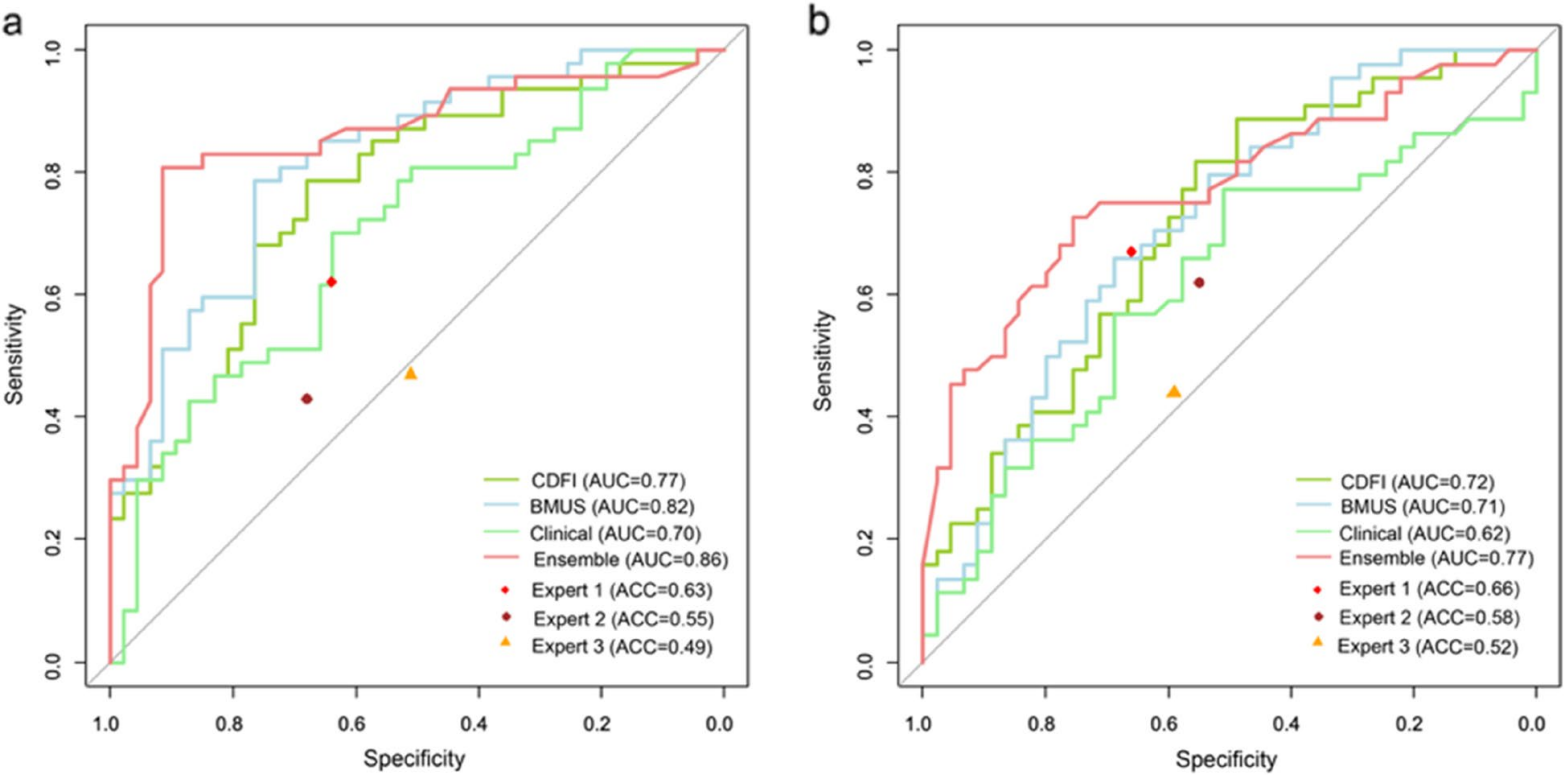
Receiver operating characteristic curves of four predictive models and expert (specificity and sensitivity) points of three radiologists for (**a**) internal test set A and (**b**) independent external test set B

The ensemble model exhibited a higher performance in predicting cases with and without CLNM from the combination of BMUS, CDFI images, and clinical variables compared with single information (Tables 2a and 3a-b and Fig. 3a). The ensemble model acquired a test accuracy of 0.79 (95% CI 0.69–0.86), F1 score of 0.79, AUC of 0.86 (95% CI 0.78–0.94), sensitivity of 0.83 (95% CI 0.72–0.94), and specificity of 0.74 (95% CI 0.62–0.87) in the primary internal test set. In addition, the DCNN model was also applied to a secondary external test set to examine the model’s generalizability (Tables 2b and 3c-d and Fig. 3b). The ensemble model achieved comparative performance on the secondary test set with an accuracy of 0.72 (95% CI 0.61–0.81), F1 score of 0.72, AUC of 0.77 (95% CI 0.68–0.87), sensitivity of 0.75 (95% CI 0.61–0.86), and specificity of 0.69 (95% CI 0.56–0.82).

**Table 2:** Performance of the four models and three radiologists according to the test sets

Modality	AUC	Accuracy	Sensitivity	Specificity	PPV	NPV	Kappa value	F1 score
a. Performance metrics of the models and US specialists on the primary internal test set A.								
Clinical	0.70 (0.59–0.80)	0.63 (0.52–0.73)	0.62 (0.49–0.74)	0.64 (0.51–0.77)	0.63	0.63	0.26	0.62
BMUS	0.82 (0.74–0.90)	0.77 (0.67–0.85)	0.81 (0.70–0.91)	0.72 (0.60–0.85)	0.75	0.79	0.53	0.78
CDFI	0.77 (0.67–0.86)	0.70 (0.60–0.79)	0.85 (0.74–0.94)	0.55 (0.40–0.68)	0.66	0.79	0.40	0.74
Ensemble	0.86 (0.78–0.94)	0.79 (0.69–0.86)	0.83 (0.72–0.94)	0.74 (0.62–0.87)	0.78	0.82	0.57	0.79
Expert 1	N/A	0.63 (0.52–0.73)	0.62 (0.46–0.75)	0.64 (0.48–0.77)	0.63	0.63	0.26	0.62
Expert 2	N/A	0.55 (0.42–0.67)	0.43 (0.29–0.58)	0.68 (0.53–0.80)	0.57	0.54	0.15	0.49
Expert 3	N/A	0.49 (0.39–0.60)	0.47 (0.32–0.62)	0.51 (0.36–0.66)	0.49	0.49	0.11	0.48
b. Performance metrics of the models and US specialists on the secondary external test set B.								
Clinical	0.62 (0.51–0.72)	0.60 (0.49–0.70)	0.66 (0.52–0.80)	0.58 (0.42–0.71)	0.60	0.63	0.24	0.63
BMUS	0.71 (0.61–0.82)	0.66 (0.54–0.75)	0.73 (0.57–0.85)	0.60 (0.44–0.74)	0.64	0.69	0.33	0.68
CDFI	0.72 (0.62–0.83)	0.67 (0.57–0.77)	0.77 (0.64–0.89)	0.58 (0.42–0.71)	0.64	0.72	0.39	0.70
Ensemble	0.77 (0.68–0.87)	0.72 (0.61–0.81)	0.75 (0.61–0.86)	0.69 (0.56–0.82)	0.70	0.74	0.44	0.72
Expert 1	N/A	0.66 (0.54–0.75)	0.67 (0.51–0.80)	0.66 (0.50–0.79)	0.67	0.66	0.33	0.67
Expert 2	N/A	0.58 (0.47–0.70)	0.62 (0.47–0.76)	0.55 (0.39–0.69)	0.58	0.59	0.17	0.60
Expert 3	N/A	0.52 (0.41–0.63)	0.44 (0.30–0.60)	0.59 (0.43–0.73)	0.53	0.51	0.03	0.48

**Table 3:** Confusion matrices of the four models and three radiologists according to the test sets

a. Confusion matrices of DCNN models on test set A								
Prediction	BMUS (truth)		CDFI (truth)		Clinical (truth)		Ensemble (truth)	
	Non-metastasis	Metastasis	Non-metastasis	Metastasis	Non-metastasis	Metastasis	Non-metastasis	Metastasis
Non-metastasis	34	9	26	7	30	18	35	8
Metastasis	13	38	21	40	17	29	12	39
b. Confusion matrices of radiologists on test set A								
Prediction	Expert 1 (truth)		Expert 2 (truth)		Expert 3 (truth)			
	Non-metastasis	Metastasis	Non-metastasis	Metastasis	Non-metastasis	Metastasis		
Non-metastasis	30	18	32	27	24	25		
Metastasis	17	29	15	20	23	22		
c. Confusion matrices of DCNN models on test set B								
Prediction	BMUS (truth)		CDFI (truth)		Clinical (truth)		Ensemble (truth)	
	Non-metastasis	Metastasis	Non-metastasis	Metastasis	Non-metastasis	Metastasis	Non-metastasis	Metastasis
Non-metastasis	27	12	26	10	26	15	31	11
Metastasis	18	32	19	34	19	29	14	33
d. Confusion matrices of radiologists on test set B								
Prediction	Expert 1 (truth)		Expert 2 (truth)		Expert 3 (truth)			
	Non-metastasis	Metastasis	Non-metastasis	Metastasis	Non-metastasis	Metastasis		
Non-metastasis	29	15	24	17	26	25		
Metastasis	15	30	20	28	18	20

### DCNN model predicts CLNM better than radiologists

We also compared the predictive performance of the DCNN model with human experts with 6 years of diagnostic experience at least. As shown in Tables 2 and 3, human experts have achieved unsatisfactory predictions of CLNM. Compared to three experts averaged, the ensemble DCNN model achieved higher test accuracy (0.79 vs. 0.56, *p* = 0.026), sensitivity (0.83 vs. 0.51, *p* = 0.011), and specificity (0.74 vs. 0.61, *p* = 0.051) for dataset A and accuracy (0.72 vs. 0.59, *p* = 0.074), sensitivity (0.75 vs. 0.58, *p* = 0.039), and specificity (0.69 vs. 0.60, *p* = 0.078) for dataset B. To make a more intuitional comparison, the points of specificity and sensitivity for three radiologists’ performance on the two test sets were plotted in the same ROC space as in Fig. 3. At the same specificity as the human panel, the ensemble artificial intelligence (AI) model achieved higher sensitivity across the two test sets. Besides, the ensemble AI model obtained higher accuracy, PPV, NPV, and F1 score compared with the radiologists’ performances. Thus, deep learning models outperformed radiologists in predicting CLNM based on primary thyroid cancer US images and clinical variables, with statistically significant differences.

### Interpretability of the DCNN model

**Fig. 4:**
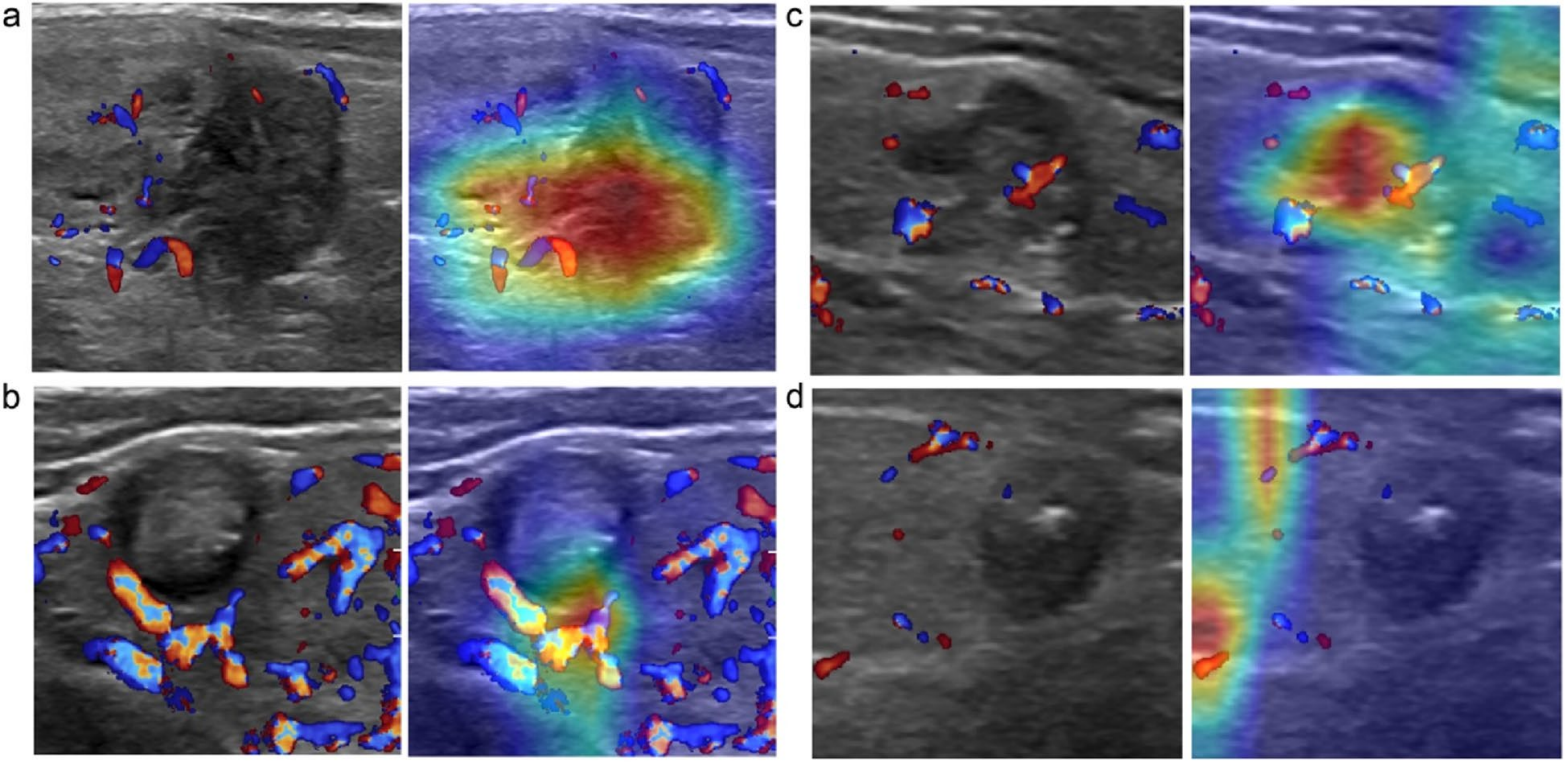
Representative US images overlaid with heat maps of four PTC patients with clinically negative lymph nodes for model interpretation, followed by (**a**) true-positive, (**b**) true-negative, (**c**) false-positive, and (**d**) false-negative examples

**Fig. 5:**
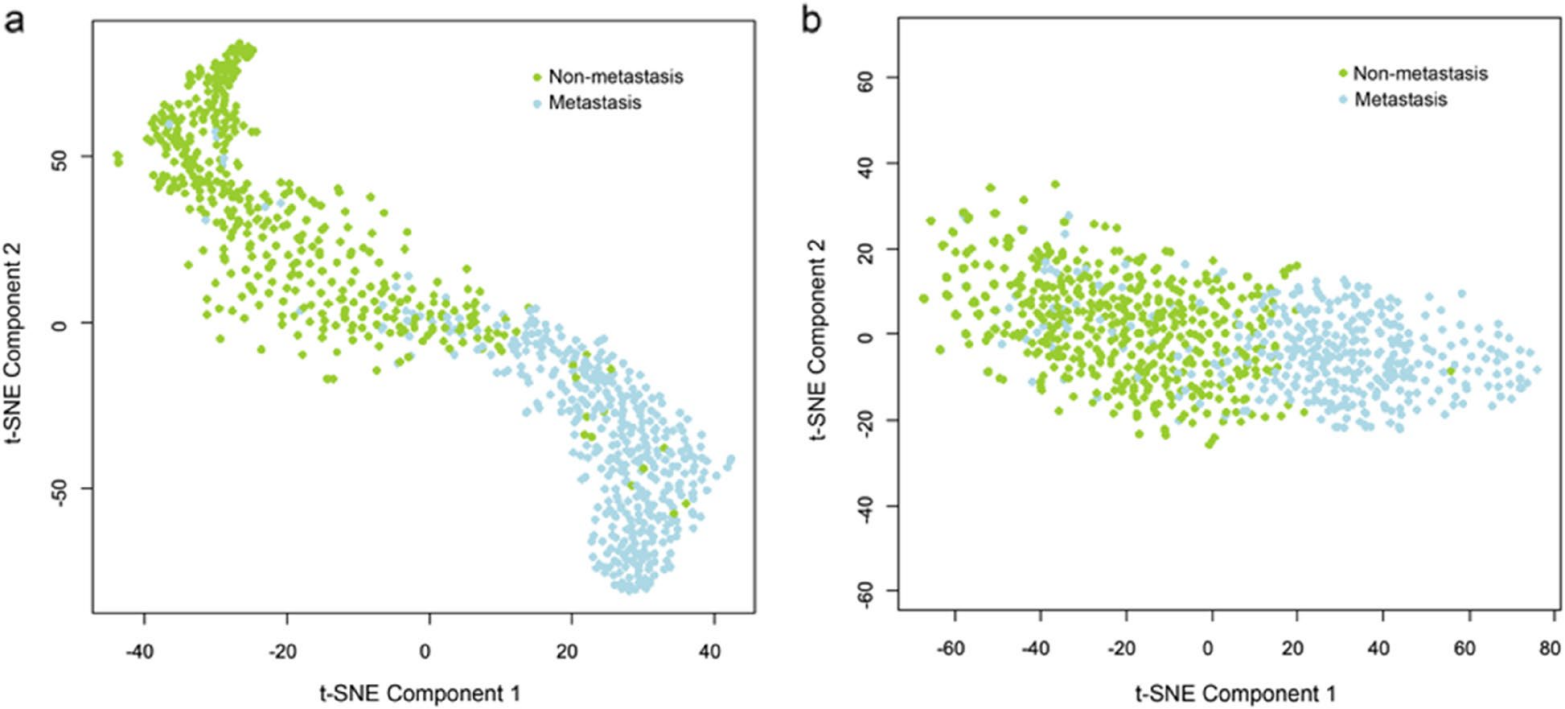
Visualization of the training set of (**a**) B-mode ultrasound (BMUS) and (**b**) color Doppler flow imaging (CDFI) after dimension reduction with t-distributed Stochastic neighbor embedding (t-SNE). Green dots represent the samples from cervical lymph node non-metastasis, and blue dots represent the samples from cervical lymph node metastasis

To better explain the AI model predictions, we utilized the approach of gradient-weighted class activation mapping (Grad-CAM) to visualize the most indicative image areas of CLN by producing heat maps [26]. The feature heat map was filtered from the last convolutional layer which was made transparent to the prediction of CLN status as shown in Fig. 4. The darker the characteristic color, the greater the possibility of CLNM, which indicates that the deep learning model focuses on the most predictive image characteristics related to CLNM. In addition, we also adopt the t-distributed stochastic neighbor embedding (t-SNE) method to illustrate the overall prediction effect by converting the representation of the last layer of the deep neural network before the prediction node for every image in the test dataset into color-coded as metastasis or non-metastasis. The results show that, compared with histopathological diagnosis, t-SNE representation of the final convolutional layer of the CNN model demonstrates favorable separation of metastatic and non-metastatic lesions (Fig. 5).

## Discussion

Building on recent developments in deep learning, in this study, we have developed an ensemble DCNN model for automatically predicting CLNM in clinically node-negative PTC patients, which directly used standard primary thyroid cancer US images and related clinical variables. Encouragingly, the resultant model performed appreciably better than radiologists for predicting CLNM in two datasets from highly distinct populations and exhibited its unique advantages in clinical diagnosis. Practically, an ideal AI model ought to be prosecuted as a predictive tool for risk stratification to help clinicians comprehend the metastatic risk of lesions. With exceptional AUC (0.86 for test A and 0.77 for test B) and higher accuracy/specificity/sensitivity compared to experts, these clinical parameters and US images combined DCNN model might have the potential to serve as an innovative CLNM predictive biomarker to reduce invasive inspections for patients with early-stage thyroid cancer.

As the first-line diagnostic procedure to evaluate the status of cervical lymph node, US offers very high diagnostic feasibility with typical features, such as rounded shape, hyperechoic, cystic degeneration, mild calcification, and peripheral vascularization, but also demonstrates restrictions in evaluating the deep anatomical space of the central cervical compartment [4, 5]. CT has been reported as an alternative to conquer the low sensitivity drawback of US diagnosis [27, 28] but also restricted by the high cost of contrast agents and stunning iodine absorption [29]. Furthermore, the absence of suspicious imaging features of CLNM for clinically node-negative thyroid cancer will further confuse the decision support to clinicians. Numerous researches have proved that several US features of primary thyroid cancer are intently associated to CLNM and have the potential to enable improvements in the preoperative evaluation of the status of lymph nodes [7, 9–12]. The risk of CLNM increased with the US size of the primary tumor (> 7 mm) [9]. Particularly, if the tumor size is larger than 4 cm, prophylactic central neck dissection (CND) was recommended to be conducted in clinically node-negative PTC by the ATA guidelines [29]. The higher vascularization degree substantially attributed to tumor metastasis to lymph nodes [30]. In addition, the existence of micro-calcifications [10], HT [7], and capsule invasion [9] detected on thyroid US images also demonstrated predictive significance for CLNM. Moreover, the shorter the distance between the tumor and the anterior and posterior borders of the thyroid capsule, the greater the risk of developing CLNM [31]. Unfortunately, such pure visual assessment that relies on experience may be personal and subjective, and radiologists are highly difficult to make accurate judgments of CLNM directly based on these US characteristics of thyroid cancer lesions.

AI has achieved considerable advancement which automatically indicates and illustrates complex data. There are two widely applied AI techniques in medical imaging at present, which are traditional machine learning and deep learning algorithms [16]. Several radiological studies have utilized random forest or support vector machine, to predict CLNM based on US radiomics [9, 15]. However, these pre-defined radiological characteristics are low throughput and exhibit low discrimination ability and reproducibility. Deep learning presents an augmentation over radiomic as it can make use of successively more abstract representations of the input data and enable augments in the decision support to clinicians [32]. Recently, Lee et al. exploited a novel computer-aided diagnosis (CAD) system containing eight deep learning models to classify CLNM in thyroid cancer on preoperative contrast-enhanced CT with the best AUC of 0.953 achieved by the ResNet50 algorithm [33] and validated the models’ diagnostic performance in a large clinical cohort with the best AUC of 0.884 acquired from the Xception algorithm [17]. However, this system is not suitable for thyroid cancer patients with clinically negative nodes. Previous studies have also suggested that deep learning could localize and differentiate the metastatic lymph nodes in US using the CNN-global average pooling (GAP) model [34]. Although the internal test accuracy was reported to be 83%, the lack of an external test set and the small cohort size make the generalization questionable.

Compared with the single deep learning architecture, the state-of-the-art ensemble deep neural network achieved superior performance on predicting lymph node metastasis as the result of combining more practical information including clinical variables, BMUS, and CDFI images. Several clinical variables, such as age, sex, and tumor maximum size, were identified as significant factors to influence the disease progression and prognosis [35]. CLNM was known to be found more frequently in younger females with larger-sized PTC patients. In addition to the typical features of visualization such as morphology, echo, and blood flow, BMUS and CDFI models can additionally extract a great quantity of detailed forecast information that is invisible to the human eyes. The potential to base decisions on multi-channel information from a single tumor could lower the challenge of tumor heterogeneity, which may be a key to improve predictive accuracy.

The ATA guidelines recommend prophylactic CND management in PTC patients with clinically negative nodes, especially those with extrathyroidal extension (ETE) or tumor size larger than 4 cm [29]. However, ongoing controversy exists since only two characteristics are far from sufficient to accurately predict precise CLNM, which may miss a large part of subclinical CLNM or lead to some unnecessary routine surgical procedures [36]. Notably, prophylactic CND does not reduce the frequency of local recurrences but improves the incidence of a series of complications, such as hypoparathyroidism and recurrent nerve injury [37]. Minimizing the incidence of local recurrence and reoperation should be weighed against the possibility of increased injury and perceived lack of benefit. Therefore, identifying more risk factors for CLNM and establishing smart risk models for stratifying PTC patients is essential to help assess prognosis and design appropriate treatment strategies. Our well-designed DCNN algorithm can accomplish this clinically meaningful purpose by predicting CLNM due to non-invasive examination, screening patients with the most likely positive lymph nodes, and minimizing the harm caused by excessive medical treatment. Automatic prediction procedures reduce human subjective intervention and facilitate clinical decision making.

A limitation of this study was that the ensemble model has not yet been tested prospectively in clinical settings and, although we are planning a randomized clinical trial, we are currently only aware of the results of a thorough retrospective test. In our study, only conventional US categories were exploited. The inclusion of advanced US information such as ARFI and SWE may further increase model accuracy. Although internal and external tests indicate good transferability between populations, the challenges related with standardization remain, as shown by the differences between US scanners. Differences between radiologists might also be seen in image handling procedures, and therefore, the standard operating procedures are recommended to promote data consistency provided by the authority. A well-known disadvantage of deep learning is its easily overfit nature, particularly when trained with a small amount of image data. We employed data augmentation and early stopping techniques to protect the model from overfitting, but great amounts of data are vital for training DCNN. Despite our best efforts to offer details of the research methodology, it may still be difficult for other researchers to replicate this research. To improve reproducibility, we make our algorithm code available on GitHub for use in other studies.

## Conclusion

In summary, a clinically advantageous predictive model for lymph node metastasis has been developed using deep learning allied to clinical variables, two-dimensional gray-scale US images, and color Doppler US images. The assay has been extensively evaluated in the internal test set and independent external test set and outperforms the performance of three experienced radiologists, which indicates that the ensemble model can potentially be an efficacious option to screening for CLNM in clinically node-negative thyroid cancer. With further optimization and calibration, it has a huge capacity to act as a powerful assistant tool to facilitate preoperative decision-making in a clinical setting.

### Supplementary Information

**Additional file 1.** 1. Introduction for the American College of Radiology Thyroid Imaging Reporting and Data System (ACR TIRADS). 2. Introduction for the scoring system.

## Data Availability

The raw data and the corresponding code have been uploaded to GitHub at https://github.com/MedicalDataAI/LNMP (ID: 8d22e54). Further information is available from the corresponding author upon request.

## Abbreviations

ATA: American Thyroid Association
BMUS: B-mode ultrasound
CDFI: Color Doppler flow imaging
CLNM: Cervical lymph node metastasis
DCNN: Deep convolutional neural network
PTC: Papillary thyroid cancer
