# Supplementary material for: Deep learning predicts cervical lymph node metastasis in clinically node-negative papillary thyroid carcinoma
Source: Insights Imaging. 2023 Dec 20;14:222. doi: 10.1186/s13244-023-01550-2 (PMC10733258; doi:10.1186/s13244-023-01550-2)
Supplement: Supplementary file 1 — Additional file 1. 1. Introduction for the American College of Radiology Thyroid Imaging Reporting and Data System (ACR TIRADS). 2. Introduction for the scoring system. [file 13244_2023_1550_MOESM1_ESM.docx]

**Deep learning predicts cervical lymph node metastasis in clinically node-negative papillary thyroid carcinoma**

**ELECTRONIC SUPPLEMENTARY MATERIAL**

**1. Introduction for the American College of Radiology Thyroid Imaging Reporting and Data System (ACR TIRADS)**

As a novel risk-stratification system for classifying thyroid nodules on the basis of US imaging, ACR TI-RADS points in five feature categories (composition, echogenicity, shape, margin, and echogenic foci) are summed to determine a risk level, including TR1 (benign, 0 points), TR2 (not suspicious, 2 points), TR3 (mildly suspicious, 3 points), TR4 (moderately suspicious, 4-6 points), and TR5 (highly suspicious, 7 points) ^[1]^. When evaluating nodules, one feature should be selected from each of the first four categories, and all features from the last category should be applied, and finally the points should be summed. It is believed that the higher the degree of malignancy of thyroid nodules, the greater the possibility of lymph node metastasis.

**2. Introduction for the scoring system**

The probability score of 1-100 for the probability of CLNM was designed to aid radiologists in making the final predictions. We collected the same amount of ultrasound images of LN metastasis and LN non-metastasis with typical characteristics and conducted a predictive training for three radiologists. Positive signs include tumor size greater than 4 cm, the coexistence of capsule invasion, micro-calcification, Hashimoto’s thyroiditis (HT), and internal vascularity. Each of these five features accounts for 20% of the overall scoring system. The radiologist makes a prediction of the likelihood of metastasis based on the ultrasound characteristics of each image. If the likelihood is greater than 50%, that is, there are more than three positive signs, then LN metastasis is considered to exist, and vice versa.

**3.** **Tables**

a. Confusion matrices of three single models on test set A

| Prediction | BMUS (Truth) | | | | CDFI (Truth) | | | | | Clinical (Truth) | | | |  |
| --- | --- | --- | --- | --- | --- | --- | --- | --- | --- | --- | --- | --- | --- | --- |
|  | nonmetastasis | | | metastasis | | nonmetastasis | | | metastasis | | nonmetastasis | | metastasis | |
| nonmetastasis | | 34 | 9 | | | | 26 | 7 | | | 30 | 18 | | |
| metastasis | | 13 | 38 | | | | 21 | 40 | | | 17 | 29 | | |

b. Confusion matrices of three single models on test set B

| Prediction | BMUS (Truth) | | | CDFI (Truth) | | | | | Clinical (Truth) | | | |  |
| --- | --- | --- | --- | --- | --- | --- | --- | --- | --- | --- | --- | --- | --- |
|  | nonmetastasis | | metastasis | | nonmetastasis | | | metastasis | | nonmetastasis | | metastasis | |
| nonmetastasis | | 27 | 12 | | | 26 | 10 | | | 26 | 15 | | |
| metastasis | | 18 | 32 | | | 19 | 34 | | | 19 | 29 | | |

**Reference:**

[1] Tessler FN, Middleton WD, Grant EG. Thyroid Imaging Reporting and Data System (TI-RADS): A User's Guide [published correction appears in Radiology. 2018 Jun;287(3):1082]. Radiology. 2018;287(1):29-36.
